# Supplementary material for: Analysis of the SARS-CoV-2 epidemic in Italy: The role of local and interventional factors in the control of the epidemic
Source: PLoS One. 2020 Nov 12;15(11):e0242305. doi: 10.1371/journal.pone.0242305 (PMC7660511; doi:10.1371/journal.pone.0242305)
Supplement: S1 Table — (DOCX) [file pone.0242305.s002.docx]

|  | Population density (persons/km^2^) | Gross domestic product *pro capite* (€) | Distance (km) |
| --- | --- | --- | --- |
| V Aosta | 39 | 35,200 | 181 |
| Trento | 87 | 30,186 | 241 |
| Lombardia | 422 | 38,200 | 1 |
| Piemonte | 172 | 30,300 | 138 |
| Liguria | 286 | 29,678 | 145 |
| Emilia Romagna | 199 | 35,300 | 210 |
| Bolzano | 72 | 42,300 | 295 |
| Marche | 162 | 26,600 | 537 |
| Veneto | 267 | 33,100 | 273 |
| Toscana | 162 | 30,500 | 299 |
| Friuli V G | 153 | 31,000 | 412 |
| Abruzzo | 121 | 24,400 | 627 |
| Umbria | 104 | 24,300 | 455 |
| Lazio | 341 | 32,900 | 586 |
| Puglia | 206 | 18,000 | 880 |
| Molise | 69 | 19,500 | 739 |
| Sardegna | 68 | 20,300 | 881 |
| Campania | 424 | 18,200 | 786 |
| Sicilia | 194 | 17,400 | 1,466 |
| Basilicata | 56 | 20,800 | 929 |
| Calabria | 128 | 17,100 | 1,157 |

**S1 Table. Local variables analyzed for the association with epidemic parameters**
