# Supplementary material for: Analysis of the SARS-CoV-2 epidemic in Italy: The role of local and interventional factors in the control of the epidemic
Source: PLoS One. 2020 Nov 12;15(11):e0242305. doi: 10.1371/journal.pone.0242305 (PMC7660511; doi:10.1371/journal.pone.0242305)
Supplement: S2 Table — (DOCX) [file pone.0242305.s003.docx]

|  | Distance (km) | Gross domestic product *pro capite* (€) | No. cases/100,000 persons at lockdown | No. swabs *per* case |  |  |
| --- | --- | --- | --- | --- | --- | --- |
| Distance (km) | - | -0,81 | -0,86 | 0,81 |  |  |
| Gross domestic product *pro capite* (€) | -0,81 | - | 0,77 | -0,57 |  |  |
| No. cases/100,000 persons at lockdown | -0,86 | 0,77 | - | -0,70 |  |  |
| No. swabs *per* case | 0,81 | -0,57 | -0,70 | - |  |  |

**S2 Table. Correlation matrix between local and interventional variables.**

Spearman correlation coefficient is shown (the minus sign indicates an inverse correlation). All correlations were significant (p<0.01).
