# Supplementary material for: Analysis of the SARS-CoV-2 epidemic in Italy: The role of local and interventional factors in the control of the epidemic
Source: PLoS One. 2020 Nov 12;15(11):e0242305. doi: 10.1371/journal.pone.0242305 (PMC7660511; doi:10.1371/journal.pone.0242305)
Supplement: Attachment — Submitted filename: Response to reviewers.docx [file pone.0242305.s004.docx]

PONE-D-20-19843 Response to Reviewers

Reviewer #1: This manuscript is about the Analysis of SARS-CoV-2 epidemic in Italy: role of baseline and intervention factors in the epidemic control.
In as much as SARS-CoV-2 manuscripts are of interest, I find almost every aspect of the paper needs further revision for clarity; context; methodological rigor; and interpretation. Please see my comments below:

Firstly. Please find native speaker to assist with the English language editing and flow of the manuscript.

R: The manuscript was revised by a native speaker

Abstract
Please give a brief introduction of what your paper seeks to achieve (aims and objectives of your paper)
Also, please talk about the methodology used. for example how your sample was achieved and how you interpreted your results. for example, talk about cases were represented in xxx per 10,000 and xxxx was shown in means, or percentages to make your work more readable to the international world.
At the bottom part of your abstract, please talk a little on conclusion.

R: we thank the Reviewer for his suggestion and revised the abstract accordingly.

Introduction
Your introduction is too scanty. though CoV-2 is a novel disease, I believe you can deliberate more on literature in both Europe and around the globe than just talking about how lock downs and restrictions were done in Italy which is already known because of news casters.
please talk about the disease, its global burden ( for example, in USA, China, Africa, etc and limit it to Italy and compare these burdens with that of Italy. compare the international strategies of other countries with what happened in Italy. Please explore more on this.

R: On the basis of the reviewer suggestion, the Introduction was improved by adding information about the disease burden throughout the world, the major clinical characteristics of the disease, and a comparison of the international containment strategies adopted, quantified according to the Oxford stringency score.

Methods
In your methods thank you for telling us about how 'Swab' reports were taken and the availability of the data. However, why did you decide to use the Gompertz growth equation ( which is a nonlinear regression model, used to describe growth curves
mostly in biology to determine growth of animals and plants, as well as the number or volume of bacteria and cancer cells?. Can you please explain further on why this is befitting for your work, or replace it with linear curves if possible. Also, please explain more on the correlations between the variables.

R: The epidemic trajectory follow a sigmoidal curve (logistic model), thus a linear regression model is not suitable for the analysis. Usually the 5-parameters Richards curve is adopted. We used a re-parametrization of the Gompertz growth equation which is a special case of the Richards equation. The Gompertz curve has a lower number of parameters than the Richards curve (3 instead of 5): thus, for the purpose of this study, the simpler model was preferred (according to both to Akaike Information Criteria and Extra-sum-of squares F test) because is easier to fit while providing an equally acceptable description of the epidemic trajectory. In addition, the parameters analysed in this study (maximum number of infected subjects and maximum growth rate) are easily obtained from the fitted curve. We explained more on it as well as on the correlation analysis in the relevant Methods paragraphs.

Table 1: please add percentages

R: percentages have been added to Table 1

You seem to be dwelling more on the GDP of the various regions, please represent that with a graph where appropriate and compare that with the various interventions within the regions in the discussion part.

R: we added a supplementary S1 Table, showing population density, distance and GDP for each Region, and S2 Table showing the correlation matrix between the variables analyzed in multiple regression (GDP, distance, no. cases at lockdown, no. swabs per case). In our study, GDP was considered as a surrogate marker of the industrial activity of the regions, which appears to have influenced the extent of the epidemic before lockdown implementation. The infection may have spread more efficiently in the areas with higher industrialization, probably because of broader social interactions and population movement associated with the industrial activities.

Discussions
please discuss you findings as in: What was the baseline, what were the intervention factors, are they attributed to GDPs of the various regions? What are the implications, and how will that be used as a future baseline implementation, should there be any future epidemic. lastly, please also discuss a bit on how the CoV-2 protocol approved by the WHO either helped or did not in the intervention factors to the control of the epidemic in Italy.
Thank You.

We thank the reviewer for the suggestions on Discussion improvement. We replaced the term “baseline” factor with “local” factors, which appears more appropriate. We defined what are the local and interventional factors considered (para 2), then we discussed about the role of local factors (para 3) and about the role of interventional factors and their association with GDP and distance (paras 4-6). In the last paras (7-9) we discussed about implications of our analysis for the management of secondary waves or future epidemic. We mentioned also the WHO interim guidance on response actions for COVID-19 both in the Introduction and in the Discussion. We believe that now the Discussion is clearer.

Reviewer #2: Thanks for developing this nice manuscript. Authors analyzed the impact of baseline and interventional variables on the epidemic curve in each region in Italy. They found that the number of cases correlated inversely with the distance from the area in which first cases were detected and directly also with the gross domestic product pro capite of the Region. In their opinion, earlier start of the lockdown and a wider testing were associated with a lower final number of total cases, which could help policy makers to act promptly.

My only comment on the analysis technique: did you consider the probability of reinfection in your model? It may be true that 99% of the population will got Covid by 113 days in Italy, but if reinfection occurs after around one month for some of the recovered patients, how we will fit that in our model? Please respond or adjust your analysis.

R: we appreciate the reviewer’s considerations about our manuscript. The model does not consider the probability of reinfection but only new cases of infection. However, there was no case of reinfection reported in Italy during the period analyzed.
